# Forecasting COVID-19 cases using time series modeling and association rule mining

**DOI:** 10.1186/s12874-022-01755-x

**Published:** 2022-11-01

**Authors:** Rachasak Somyanonthanakul, Kritsasith Warin, Watchara Amasiri, Karicha Mairiang, Chatchai Mingmalairak, Wararit Panichkitkosolkul, Krittin Silanun, Thanaruk Theeramunkong, Surapon Nitikraipot, Siriwan Suebnukarn

**Affiliations:** 1 College of Digital Innovation Technology, Rangsit University, Pathum Thani, 12000 Thailand; 2 Faculty of Dentistry, Thammasat University, Pathum Thani, 12121 Thailand; 3 Faculty of Engineering, Thammasat University, Pathum Thani, 12121 Thailand; 4 Faculty of Medicine, Thammasat University, Pathum Thani, 12121 Thailand; 5 Faculty of Science and Technology, Thammasat University, Pathum Thani, 12121 Thailand; 6 Sirindhorn International Institute of Technology, Thammasat University, Pathum Thani, 12121 Thailand; 7 Academy of Science, Royal Society of Thailand, Sanam Sueapa, Khet Dusit, Bangkok, 10300 Thailand; 8 Thammasat University Hospital, Pathum Thani, 12121 Thailand; 9 Research and Innovation Division, Thammasat University, Pathum Thani, 12121 Thailand

**Keywords:** COVID 19, Pandemic, Data mining, Time series analysis, Association rule mining

## Abstract

**Background:**

The aim of this study was to evaluate the most effective combination of autoregressive integrated moving average (ARIMA), a time series model, and association rule mining (ARM) techniques to identify meaningful prognostic factors and predict the number of cases for efficient COVID-19 crisis management.

**Methods:**

The 3685 COVID-19 patients admitted at Thailand’s first university field hospital following the four waves of infections from March 2020 to August 2021 were analyzed using the autoregressive integrated moving average (ARIMA), its derivative to exogenous variables (ARIMAX), and association rule mining (ARM).

**Results:**

The ARIMA (2, 2, 2) model with an optimized parameter set predicted the number of the COVID-19 cases admitted at the hospital with acceptable error scores (*R*^2^ = 0.5695, RMSE = 29.7605, MAE = 27.5102). Key features from ARM (symptoms, age, and underlying diseases) were selected to build an ARIMAX (1, 1, 1) model, which yielded better performance in predicting the number of admitted cases (*R*^2^ = 0.5695, RMSE = 27.7508, MAE = 23.4642). The association analysis revealed that hospital stays of more than 14 days were related to the healthcare worker patients and the patients presented with underlying diseases. The worsening cases that required referral to the hospital ward were associated with the patients admitted with symptoms, pregnancy, metabolic syndrome, and age greater than 65 years old.

**Conclusions:**

This study demonstrated that the ARIMAX model has the potential to predict the number of COVID-19 cases by incorporating the most associated prognostic factors identified by ARM technique to the ARIMA model, which could be used for preparation and optimal management of hospital resources during pandemics.

## Background

The crisis outbreak of coronavirus disease 2019 (COVID-19) caused by severe acute respiratory syndrome coronavirus 2 (SARS-CoV-2) started in Wuhan, Hubei Province, China in December 2019 [1]. The COVID-19 pandemic has required governments around the world to implement new policies under pressure from vulnerable people and communities [2]. Since the first outbreak, COVID-19 has mutated into many variants including the alpha, beta and delta SARS-COV-2 variants, which have been associated with new waves of infection [3]. The catastrophic effect across the entire world resulted in more than six million deaths worldwide in 2022 [4]. In addition, COVID-19 has caused a rapid deterioration in the condition of the disease, and the number of patients requiring hospitalization has increased significantly, resulting in a high demand for hospital resources [1].

Data mining is an efficient analytical methodology to recognize and investigate a huge data set to acquire meaningful information [5]. In the medical field, the large numbers of medical records (including demographic information, diagnoses, clinical notes, etc.) in the healthcare information systems are ideal targets for the use of data mining in improving the analysis and prognosis prediction of various diseases [6–8]. Examples include using an Artificial Neural Network (ANN) and Support Vector Machine (SVM) algorithm to predict cardiovascular disease [9], using data mining classification algorithms, Decision Tree and Naive Bayes algorithms to identify liver disease [10] and predict the recovery outcome of Middle East Respiratory Syndrome Coronavirus (MERS-CoV) [11]. With the unprecedented increase in COVID-19 cases worldwide, there is a need for effective prediction models to identify the associated prognostic factors and forecast the number of COVID-19 cases to optimally organize the hospital resources.

Time series analysis and association rule mining (ARM) models have been widely used to predict trends, structural breaks, cycles, and unobserved values, and have proven to be useful in the medical field [12–14]. The auto regressive integrated moving average (ARIMA), a time series analysis model, was shown to have a promising accuracy for forecasting of infectious diseases in medical fields [15, 16]. ARIMA was used to forecast the number of new COVID-19 cases, deaths, and recoveries based on the daily reported data from different countries for assessment of the future outbreak [17–20]. ARM was originally presented by Agrawal et al. as an algorithm for marketing data analysis [21]. ARM has been used to extract medical health information, which is currently being applied for the development of classification and prediction models to identify and forecast the possibility of development and progression of a disease by considering the rules of the disease [22]. ARM was demonstrated to be an effective model for mining the frequent symptom pattern for COVID-19 patients, which could assist clinicians in decision making [23]. Another study used ARM to analyze the patterns of different non-pharmaceutical interventions to manage the infection growth rate in the United States [24]. Even though there are many advanced data-driven time series methods used to predict the future number of COVID-19 patients, a new and more accurate prediction model is important in the pandemic crisis. The associated contributing factors should be considered to improve model performance. Therefore, the combination of ARM and ARIMA models by selecting the most associated prognostic rules and integrating with ARIMA models could increase the accuracy of predicting new cases to better understand the current situation and the progression of COVID-19, which can be easily used by society, organizations, or governments to assess and manage the crisis during the future outbreak.

The aim of this study was to evaluate the most effective combination of ARM techniques and ARIMA models to identify prognostic factors and predict the number of COVID-19 patients. These models are expected to allow for better preparation, organizing hospital resources of further such units and more optimal use of medical personnel and equipment to enhance healthcare decision-making to manage COVID-19 patients in this crisis situation.

## Methods

### Administration protocol and data collection

The study was conducted at Thailand’s first university-based field hospital. The field hospital was transformed from the service apartment style 14-story building of the university dormitory into a 494-bed facility for non-critical COVID-19 patients [25]. The field hospital was managed by the main university hospital and included the patients referred from the project’s five university hospitals and hospitals in the central area of Thailand. Sources of funding come mainly from the donations of university alumni, community groups and non-governmental organizations. Upon admission, a nurse records patient data in the COVID-19 screening of the field hospital information system; the patient undergoes a chest x-ray, blood tests for complete blood count (CBC), liver function tests (LFTs), electrolyte, balance urine nitrogen (BUN), and Creatinine (Cr). The doctor interprets the labs and chest x-ray, and records the results in the admission note. The patients are only admitted to the field hospital if they meet all of the following criteria: 1) asymptomatic, mild or moderate symptoms; 2) normal activities of daily living; 3) no important organ dysfunction; 4) no psychiatric history; and 5) resting pulse oxygen saturation (SpO_2_) > 95%. To avoid unnecessary contact between patients and medical personnel, the patient reports signs and symptoms, wants and needs via an internal field hospital application. Any consultation with the attending physician is done through a notification form. If the attending physician wishes to speak to the patient, the patient’s telephone number is obtained from the respective patient’s floor. All prescriptions must be made using a prescription form which will then be processed by the attending nurse and recorded in the progress note in the field hospital information system and in the university hospital electronic medical record system. In this field hospital system, the laboratory and radiographic examination would be performed on symptomatic COVID-19 patients with a history of taking Favipiravir and for severity assessment of symptomatic COVID-19 patients.

For Favipiravir-naive patients: 1) A follow-up chest x-ray may be considered in patients with worsening signs and symptoms (body temperature (BT) > 38.0 °C, cough, fatigue, SpO2 < 96%, or decreased SpO2 > 3% after a stress test); and 2) if the chest x-ray infers pneumonia with respiratory signs and symptoms (as mentioned in 1), refer the patient to the originating hospital for continued treatment with Favipiravir.

For patients previously treated with Favipiravir: 1) Follow-up by chest x-ray, LFTs); 2) if LFTs increase, consider consulting an ID specialist to terminate/adjust medication use; and 3) if the chest x-ray infers a progression of the infiltration accompanied by respiratory signs and symptoms (cough, fatigue, SpO2 < 96% and SpO2 drop > 3% after a stress test), consider referring the patient to the hospital of origin.

Asymptomatic patients who have been hospitalized for at least 14 days after a positive COVID-19 testing will be discharged home. The patients who received Favipiravir should fulfil all the following criteria: 1) The patients signs and symptoms have improved without progression of infiltration on chest x-ray; 2) BT < 37.8 °C continuously for 24–48 hours; 3) respiratory rate (RR) < 20/min; and SpO2 > 96% at rest. In the event of a patient’s condition deteriorating, they are quickly transferred to the designated higher-level hospitals.

The criteria for transfer are 1) meeting the criterion of severe or critical, and 2) lung imaging showing a greater than 50% progression of lesions. Patients do not need Real-time Polymerase Chain Reaction (RT-PCR) or Antigen/Antibody detection for COVID-19 prior to discharge. One day before discharge, the attending nurse informs the attending physician of the number of potential discharges, so that the physician can prepare medical certificates and insurance documents according to the patient’s needs. Upon discharge, the attending physician updates the patient’s progress and discharge summary in the electronic medical record system of the university hospital.

A total number of 3685 patient records were retrieved from the electronic hospital information systems of the referral hospitals and the field hospital information system. In this study, we included all patients confirmed with asymptomatic and mild-to-moderate COVID-19 conditions from March 2020 to August 2021 (four waves of COVID-19 in Thailand). Collected data included patient demographics, comorbidities, body mass index (BMI), job, place of exposure to coronavirus, symptom before field hospital admission, sign of pneumonia in chest x-ray, field hospital length of stay, and the field hospital discharge destination. Table 1 shows the preliminary analysis of the dataset, including attributes, values, and frequency of each attribute-value pair.

**Table 1:** Preliminary analysis of the dataset: attributes, values, and frequency of each attribute-value pair

No	Attribute name	Attribute value	Attribute code	Frequency
1	Gender	Male	sex_male	1711
		Female	sex_female	1974
2	Age (year)	Less than 24	age_24	1148
		25–44	age_45_44	1838
		45–64	age_45_64	625
		More than 65	age_65	74
3	Body Mass Index	Less than 25	bmi_25	2309
		25–29	bmi_25_29	931
		More than 30	bmi_30	445
4	Underlying	None	ud_none	3392
	Diseases	Respiratory	ud_repp	82
		Hypertension	ud_ht	39
		Metabolic	ud_meta	53
		Dyslipidemia	ud_dlp	14
		Other	ud_oth	64
		Diabetes mellitus	ud_dm	18
		Pregnant	ud_preg	23
5	Job	General worker	job_gen	3592
		Healthcare worker	job_health	93
6	Source of infection	Community	source_com	3119
		Family	source_fam	475
		Hospital	source_hosp	91
7	Symptom	Asymptomatic	symp_ast	2295
		Mild	sym_mild	1371
		Moderate	sym_mode	19
8	Chest X-ray	No lesion	cxr_no	3213
		Pneumonia	cxr_pneu	472
7	Length of stay (Day)	Less than 14	los_1_14	3625
		More than 14	los_15	60
8	Patient Discharge	Home discharge	dc_home	3600
		Refer to general hospital	dc_hosp	85
9	Current Incidence	Wave 1 (MAR-MAY 2020)	wave_1	55
		Wave 2 (JAN-MAR 2021)	wave_2	311
		Wave 3 (APR-MAY 2021)	wave_3	1779
		Wave 4 (JUN-JUL 2021)	wave_4	1540

### Time-series analysis and association analysis

**Fig. 1:**
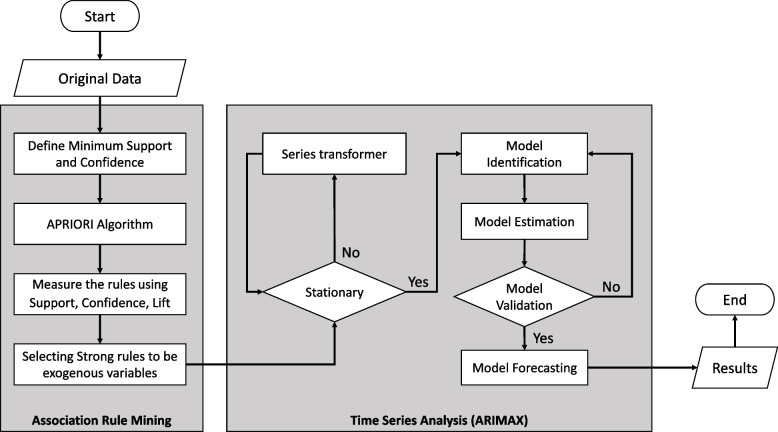
The summary of the time series and association analysis

In this work, we present a study to combine time series analysis and association analysis to forecast the COVID-19 admitted cases as well as to analyze their potential factors and characteristics. To estimate the number of new cases and to predict the prognosis for better understanding of the current situation and progression of COVID-19, we exploited the autoregressive integrated moving average (ARIMA) model and its subclasses (i.e., AR, MA, ARMA) [12, 17, 26], and association rule mining (ARM) [21, 24] as tools for investigation (Fig. 1).

### The autoregressive (AR) model

In the AR model, the predictive value at the time period t is modeled by the observed values at various time slots *t* − 1*, t* − 2*,. . ., t* − *k*. The impact of the value at each previous time period on the value at the current time is determined by the coefficient factor at that particular period of time. With this assumption, the model performs the regression of past time series and then calculates the present or future values in the series, commonly known as an auto regression (AR) model. It can be modeled as follows.

$${y}_t={\beta}_0+{\beta}_1{y}_{t-1}+{\beta}_2{y}_{t-2}+\dots +{\beta}_p{y}_{t-p}+{\varepsilon}_t$$

Here, *y*_*t*_ is the value at the current time *t*, and *y*_*t* − 1_, *y*_*t* − 2_, …, *y*_*t* − *p*_ are the observed values at the previous *p* time spots with their corresponding coefficients *β*_1_, *β*_2_, …, *β*_*p*_, respectively, *β*_0_ is the intercept, and *ε*_*t*_ is the residual error at the time *t*. Therefore, *y*_*t*_ − *ε*_*t*_ is the expected value at the current time *t*. In this work, the value *y*_*t*_ can be modeled as the number of inpatients, incoming patients, or outgoing patients at the time period *t*.

### The moving-average (MA) model

Since the value of the time period *t* may be impacted by unexpected external factors, i.e., noises, we can alleviate such impact by means of the moving average method. Analogous to AR, the predicted value at the time period *t* can be modeled by the previous *q* lagged forecast errors *ϵ*_*i*_ as follows.

$${y}_t={\phi}_0+{\phi}_1{\varepsilon}_{t-1}+{\phi}_2{\varepsilon}_{t-2}+\dots +{\phi}_q{\varepsilon}_{t-q}+{\varepsilon}_t$$

Here, *y*_*t*_ is the value at the current time *t* and the lagged errors *ε*_*t* − 1_, *ε*_*t* − 2_, …, *ε*_*t* − *q*_ are residual errors of the *q* autoregressive models at time *t* − 1 to *t* − *q* with *ϕ*_1_, *ϕ*_2_, …, *ϕ*_*q*_ as their corresponding coefficients, *ϕ*_0_ is the intercept, and *y*_*t*_ is the residual error at the time *t*. The residual error at the time points after *t* − 1 can be derived by the auto-regressive (AR) model as follows.

$${\displaystyle \begin{array}{c}{\varepsilon}_{t-1}={y}_{t-1}- \left({\beta}_0+{\beta}_1{y}_{t-2}+\cdots +{\beta}_p{y}_{t-p-1}\right. \\ {}{\varepsilon}_{t-2}={y}_{t-2}-\left({\beta}_0+{\beta}_1{y}_{t-3}+\cdots +{\beta}_p{y}_{t-p-2} \right. \\ {}\cdots \kern0.5em \cdots \\ {}{\varepsilon}_{t-q}={y}_{t-3}-\left({\beta}_0+{\beta}_1{y}_{t-q-1}+\cdots +{\beta}_p{y}_{t-p-q}\right. \end{array}}$$

Although the standard AR and MA may use the auto-correlation function (ACF), which takes into account all of the points, it is possible to apply the partial auto-correlation function (PACF), which accounts for the values of the intervals between.

### The autoregressive moving average (ARMA) model

The Auto Regressive Moving Average Model (ARMA) combines the AR and MA models. In ARMA, the impact of previous lags along with the residuals is considered for forecasting the future values of the time series as follows.

$${y}_t={\beta}_0+{\beta}_1{y}_{t-1}+{\beta}_2{y}_{t-2}+\dots +{\beta}_p{y}_{t-p}+{\phi}_1{\varepsilon}_{t-1}+{\phi}_2{\varepsilon}_{t-2}+\dots +{\phi}_q{\varepsilon}_{t-q}+{\varepsilon}_t$$

Here, *β*_*i*_ represents the coefficients of the AR model, *ϕ*_*i*_ represents the coefficients of the MA model, and *ε*_*t*_ is the residual error at the time *t*. We assume only one significant value from the AR model and one significant value from the MA model, so the ARMA model will be obtained from the combined values of these two models, denoted as the order of ARMA (1,1).

### The autoregressive integrated moving average (ARIMA) model

As a generalization of AR, MA, and ARMA, the ARIMA model introduced differencing (integration) into the ARMA model to make the series stationary exploit to forecast future values under the factor of previous lag value and residuals errors. Besides manipulating the time lag and alleviating noise by smoothing, it is also possible to decompose a series into trend, seasonal, and residual components, by assuming an additive model. With this addition, the series can be transformed to a stationary time series. To achieve the transformation, the differencing method is applied. For example, we can subtract the *t* − 1 value from *t* values of time series. After applying the first differentiation, if we are still unable to get the stationary time series, we can again apply the second-order differentiation. The ARIMA model is an extension of the ARMA model by the fact that it includes one more factor known as integrated (i.e., differentiation) which stands for *I* in the ARIMA model. The ARIMA model, denoted by ARIMA (*p,d,q*), can be formulated as follows:

$$y^{\prime}_t=\beta_0+\beta_1y^{\prime}_t+\beta_2y^{\prime}_{t-2}+\dots+\beta_py^{\prime}_{t-p}+\phi_1\varepsilon_{t-1}+\phi_2\varepsilon_{t-2}+\dots+\phi_q\varepsilon_{t-q}+\varepsilon_t$$

Here, *p* is the order of the autoregressive process, *d* (set to 1 in this case) is the degree of differentiation (the number of times the series was differenced), and *q* is the order of the moving average component. In this model, the first-order difference (*d* = 1) between consecutive observations *y*′_*i*_ was computed and used, instead of the original observed value *y*_*i*_ as shown below.

$$y^{\prime}_i=y^{\prime}_i-y^{\prime}_{i-1}$$

Differencing removes the changes in the level of a time series, eliminating trend and seasonality and, consequently, stabilizing the mean of the time series.

In some situations, we may need to difference the series data a second time (d = 2) to obtain a stationary time series, which is referred to as second order differencing as follows:

$${y}_i^{\prime\prime}={y}_i^{{\prime}}-{y}_{i-1}^{{\prime}} {y}_i^{\prime\prime}=\left({\textrm{y}}_t-{\textrm{y}}_{t-1}\right)-\left({\textrm{y}}_{t-1}-{y}_{t-2}\right) {y}_i^{\prime}={y}_t-2{y}_{t-1}+{y}_{t-2}$$

A higher-order differentiation can be pursued analogously in the same manner.

### The autoregressive integrated moving average with exogenous covariates (ARIMAX) model

When an ARIMA model includes other time series as input variables, the model is referred to as an Autoregressive Integrated Moving Average with Exogenous Covariates (ARIMAX) model. An ARIMAX model can be viewed as a multiple regression model that takes the impact of covariates on the forecasting into account, improving the comprehensiveness and accuracy of the prediction. The ARIMAX(*p,d,q*) extends the ARIMA(p,d,q) model by including the linear effect that one or more exogenous series has on the stationary response series *y*_*t*_. This method is suitable for forecasting when data is stationary/non-stationary, and multi-variate with any type of data pattern, i.e., level/trend/seasonality/cyclicity. The ARIMAX(p,d,q) model can be formulated as follows:

$${y}_t^{{\prime}}={\beta}_0+{\beta}_1{y}_{t-1}^{{\prime}}+{\beta}_2{y}_{t-2}^{{\prime}}+\cdots +{\beta}_p{y}_{t-p}^{{\prime}} +{\phi}_1{\varepsilon}_{t-1}+{\phi}_2{\varepsilon}_{t-2}+\cdots +{\phi}_q{\varepsilon}_{t-q}+{\varepsilon}_t +{\theta}_1{\left({X}_1\right)}_t+{\theta}_2{\left({X}_1\right)}_t+\cdots +{\theta}_m{\left({X}_m\right)}_t+{\varepsilon}_t$$

Here, *d* is set to 1, (*X*_*i*_)_*t*_ is the value at the time *t* of the *i -* th exogenous covariable (*X*_1_), *θ*_*i*_ is the corresponding coefficient for the covariable *X*_*i*_, and m is the number of exogenous covariables to be considered, while *p, d*, and *q* indicate the same parameters as in the ARIMA model.

### Association rule mining

Besides the time-series analysis, association rule mining (ARM) can be used as a multivariate analysis to help us understand the correlation among factors [24]. Given a dataset containing a collection of records or transactions, each record comprises a set of categorical attributes. An association rule can be denoted by *A → B*, where *A* (the antecedent or LHS) and *B* (the consequent or RHS) are sets of various attribute-value pairs (also called itemsets), and are disjoint. The rule represents the hypothesis that when variables in *A* occur in the dataset, the variables in *B* also occur. Association mining generates a large number of rules from a given dataset. In a dataset with m attributes *n* − 1 antecedents and one consequent, each with n values, each can generate a maximum of *nm*^*n* − 1^ − 1 rules. However, not all rules are significant. The goal of this approach is to find rules that have high practical significance. To eliminate spurious rules, we use three measures: support, confidence, and lift. In addition, we also use the chi-squared test to measure the statistical significance of the association between the antecedent and the consequent. Given two disjoint sets of attribute-value pairs *A* and *B*, and an association rule *A → B*; support of the rule refers to the number of records where the attribute-value pairs in either set *A* or *B* appear in the dataset relative to the total number of records (transactions or instances). This denotes the prevalence of the rule in the dataset. By definition, the support value is symmetric, that is Support (*A → B*) = Support (*B → A*), and it equals the total numbers of records containing both *A* and *B* to the total number of records in the dataset. The confidence of the rule *A → B* measures the conditional probability of *B*, given A. Thus, the confidence measure for a given rule is asymmetric, that is Confidence (*A → B*) ≠ Confidence (*B → A*). The lift measure is the ratio between the observed support and the expected support between the independent variables *A* and *B*. Implicitly, lift > 1 means a greater degree of dependence, lift < 1 specifies negative dependence, and lift = 1 indicates independence between *A* and *B*. Lift is also a symmetric measure between the itemsets A and B, that is Lift (*A → B*) = Lift (*B → A*).

$$\begin{aligned}Support\left(A\to B\right)=\frac{\left|A\cap B\right|}{N}\\ {} Confidence\left(A\to B\right)=\frac{\left|A\cap B\right|}{\left|A\right|}\\ {} Lift\left(A\to B\right)=\frac{\left|A\cap B\right|\times N}{\left|A\right|\left|B\right|}\end{aligned}$$

Here, *|A|* and *|B|* are the numbers of records that include *A* and *B*, respectively, while ∣*A* ⋂ *B*∣ is the number of records that contain both *A* and *B*. In this paper, the antecedent *A* can be either patient demo-graphics (either male or female), age (< 24, 25–44, 45–64, and > 65), body mass index or BMI (< 25, 25–29, and > 29), underlying diseases (none, respiratory, hypertension, metabolic, dyslipidemia, diabetes mellitus, pregnant, or others), job (healthcare or non-healthcare patient), inflection source (community inflection, family inflection, or hospital inflection), symptoms before field hospital admission (asymptomatic, mild, or moderate), sign of pneumonia in chest x-ray (no lesion or pneumonia) or length of stay in the field hospital (14 or > 14), and patient discharge (home discharge or refer to general hospital), as the contributing factors. On the other hand, for the consequent *B* we focus on (1) the length of stay (either 1–14 or > 14), (2) the patient discharge (either home discharge or hospital discharge), (3) the chest x-ray result, and (4) current incidence (wave 1, 2, 3 or 4). Since one assumption for ARM is that all the values of attributes are discrete, we translate the numerical data used in the study into discrete labels, as well as split the continuous data of infection growth curve into four phases.

### Experiment settings

#### Data collection and parameter settings

The dataset includes 3685 records registered with the electronic hospital information systems of the field hospital during March 2020 to August 2021. It displays characteristics of the dataset, including, attributes, values, and frequency of each attribute-value pair. Each of the nine attributes contains 2–8 possible values. Most attributes have imbalanced numbers in their values, except gender (Table 1). In our time series analysis, the target of prediction is the number of patients in the field hospital for each day during the observation period, that is March 2020 to August 2021. We have explored the value of the three ARIMA parameters as *p* ∈ {1, 2, 3}, *d* ∈ {1, 2}12, *q* ∈ {1, 2, 3} due to our preliminary test. In addition, we applied association rule mining to find the most influential factors among the eleven factors, that is patient demographics, age, body mass index, underlying diseases, job, inflection source, symptom before field hospital admission, sign of pneumonia in chest x-ray, length of stay in the field hospital, patient discharge, and current incidence. As an ARIMAX model, we extend the ARIMA(*p,d,q*) model to include the parameters as a series that are the most influential to the prediction of the number of patients in the hospital. The parameters included are known as exogenous series that are expected to trigger the stationary response on the series that we are predicting.

#### Performance metrics and evaluation

Given a data set has n values, denoted by *y*_*1*_*,*. *.*., *y*_*n*_*,* each associated with a predicted value *f*_*1*_*,. .., f*_*n*_*,* the following three metrics can be formulated. Coefficient of determination (R^2^) is the proportion of the variation in the dependent variable that is predictable from the independent variable(s) as follows:

1 $${R}^2=1-\frac{SS_r}{SS_t}$$

2 $${SS}_r=\sum\nolimits_{i}{\left({y}_i-{f}_i\right)}^2=\sum\nolimits_{i}{e}_i^2$$

3 $${SS}_t=\sum\nolimits_{i}{\left({y}_{i}-\overline{y}\right)}^2$$

4 $$\overline{y}=\frac{1}{n}\sum\nolimits_{i}{y}_i$$

Here, *SS*_*r*_ is the sum of squares of residuals, *SS*_*t*_ is the total sum of squares, proportional to the variance of the data, and $\overline{y}$ is the mean of the observed data. Ranging from 0 to 1, it provides a measure of how well observed outcomes are replicated by the model. The higher the coefficient value is, the closer the dependent variable and independent variable are.

Root mean square error (RMSE) the standard deviation of the prediction errors [27], which are a measure of the distance of the data from the regression line, indicating the concentration of the data around the line of best fit as follows:

5 $$RMSE=\sqrt{SS_r}=\sqrt{\frac{1}{2}\sum\nolimits_{i}{\left({y}_i-{f}_i\right)}^2}$$

It expresses the dispersion of these errors.

Mean absolute error (MAE) allows measurement of the average magnitude of the errors for a set of predictions, regardless of their direction.

6 $$MAE=\frac{1}{n}\sum\nolimits_{i}| {y}_i-{f}_i|$$

It represents the mean of the absolute difference in the sample between the prediction and the actual observation, taking into account that all individual differences are of equal significance. Therefore, compared to RMSE, MAE is less sensitive to outliers.

## Results

### Time series analysis

**Fig. 2:**
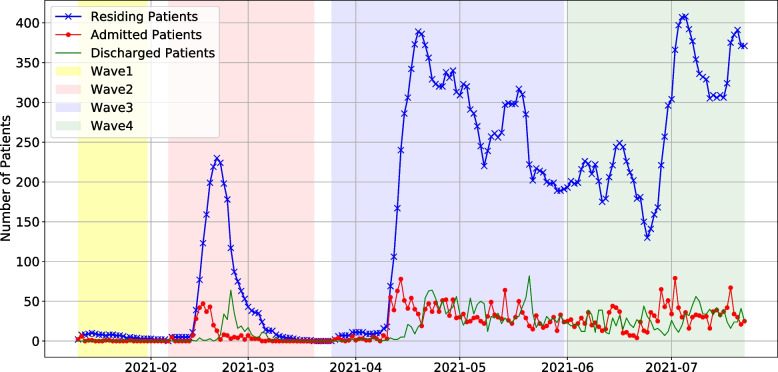
The number of daily data of patients in the field hospital; New patients; Admitted Patients; Discharged Patients in four waves of COVID-19 pandemics in Thailand

This section presents a time series analysis to forecast the number of patients admitted to the field hospital. Figure 2 shows the number of patients from 26 March 2020 to 22 July 2020. Three time series represent the relationships among a number of residing patients that are equal to a cumulative difference between admitted and discharged patients living in the hospital. The graph presents four waves of pandemic following the number of patients in hospital. The four waves are as follows: The first wave (Wave 1), the emergence of SAR-CoV-2, is the smallest period (34 days) from 26 March 2020 to 16 May 2020. The second wave (Wave 2) was from 11 January 2021 to 14 March 2020 (44 days). After that, the third wave (Wave 3) and fourth wave (Wave 4) were the continuous periods from 11 April 2021 to 31 May 2021 (51 days) and 1 June 2021 to 22 July 2021 (52 days), respectively. Finally, the forecasting models are validated by a test dataset from 1 August 2021 to 30 August 2021(30 days).

In this study, the time series models were trained using six training datasets. The first training set (All Wave) covers all datasets Wave 1 to Wave 4 of 228 days; the second training set, Wave 1 of 34 days; the third training set, Wave 2 of 45 days; the fourth training set, Wave 3 of 51 days; the fifth training set, Wave 4 of 52 days; the sixth training set, Wave 3 and Wave 4 of 103 days.

**Fig. 3:**
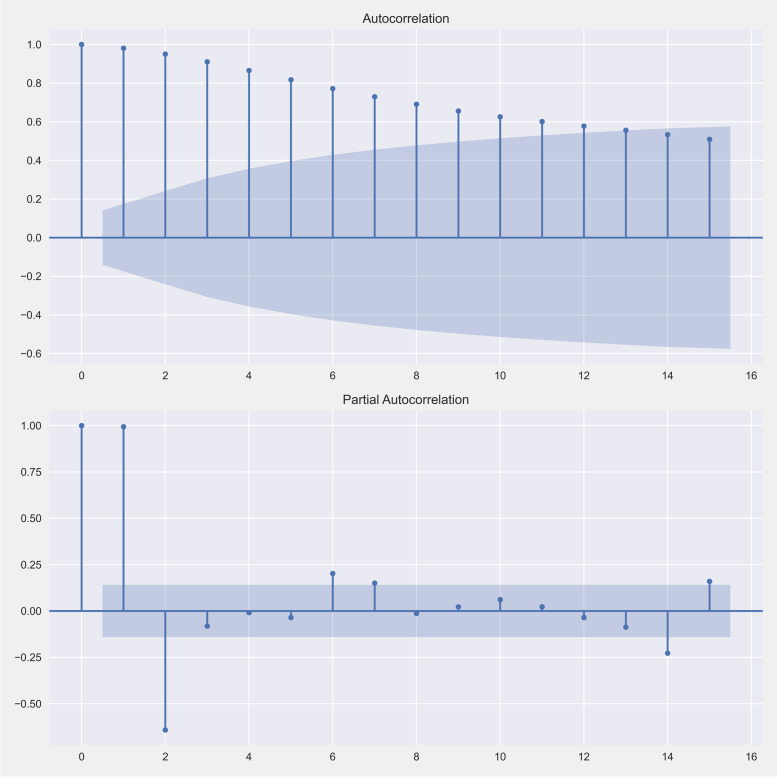
An autocorrelation function (ACF) and a partial autocorrelation function (PACF) are presented to confirm the steady-state prediction of time-series models

In this work, we tested the estimated model using an autocorrelation function (ACF) and a partial autocorrelation function (PACF) plots to ensure that the model fits the data [17]. Figure 3 presents the steady-state prediction of time-series models. An estimation of the model explored the coefficient (Coef.), the standard error (Std err.) and z. An estimate of the first model was the AR model which gave a coefficiency of 0.3808, standard error of 0.243 and z of 1.565. The second model was an MA model which gave coefficiency of − 0.5287, standard error of 6.841 and z of − 0.077. The sigma value or constant value was coefficiency of − 0.5287, standard error of 6.841 and z of − 0.077. Moreover, we further estimated the model with Jarque-Bera of 7.70, heteroskedasticity of 0.57 and skew of 0.68.

For the data set, the time series method was applied using Python (PyFlux library) for time series analysis and prediction to compare the criteria of each setting. The ARIMAX (*p,d,q*) + *X* models were parameterized with *X* ∈ {ϕ, *x*_1_, *x*_2_}, *p* ∈ {0, 1, 2, 3}, *q* ∈ {0, 1, 2, 3}, *d* ∈ {0, 1, 2}, where *X* is additional exogenous variables, with 51 combinations. Moreover, we select key features from association rule mining such as symptoms, age, and underlying diseases, etc. *X* = ϕ specifies no additional exogenous variable used. *X* = *x*_1_ indicates additional exogenous variables. There are 15 variables, composed of three attributes in the symptom feature, four attributes in the age feature, and eight attributes in the underlying diseases feature. *X* = *x*_2_ represents four variables of the selected attributes, that is the ‘moderate’ symptom, the ‘more-than-65’ age, and the underlying diseases of ‘diabetes mellitus’ and ‘pregnant.’

**Fig. 4:**
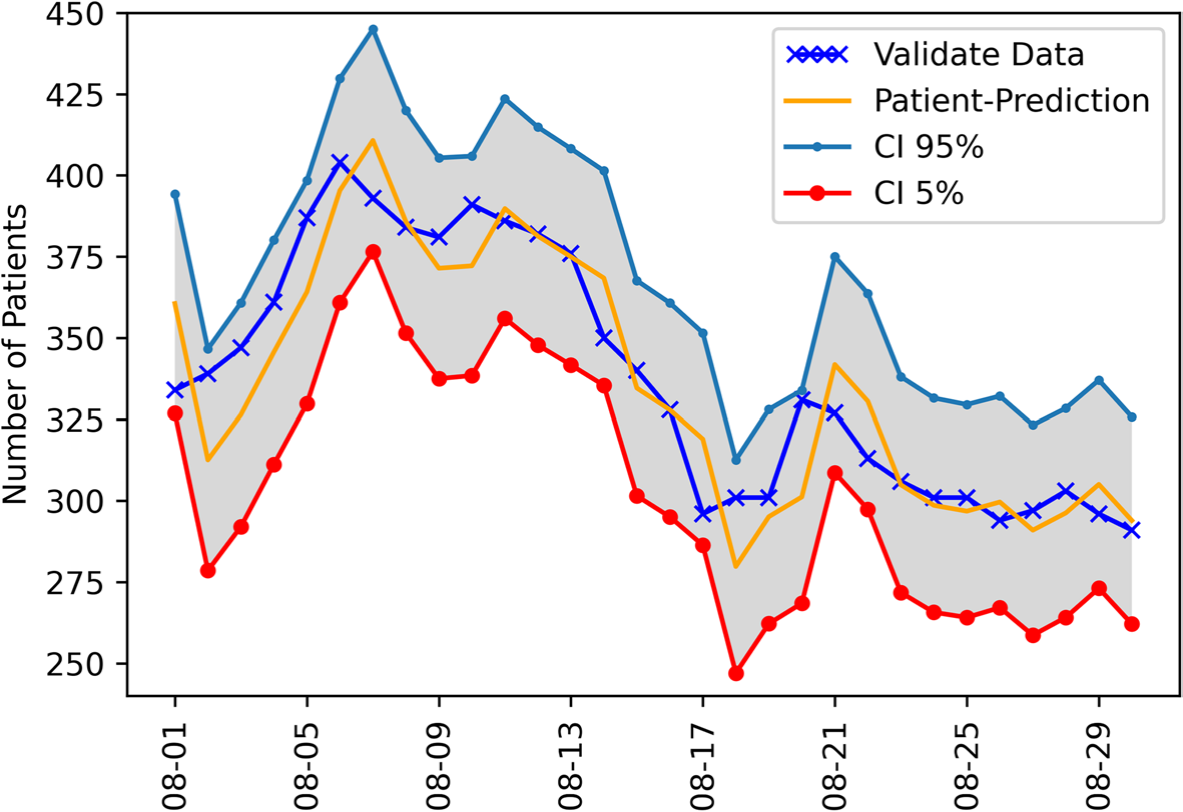
The ARIMA (2,2,2) forecasting value of the admitted patients with prediction confidential intervals (CI) between 5 and 95%

**Fig. 5:**
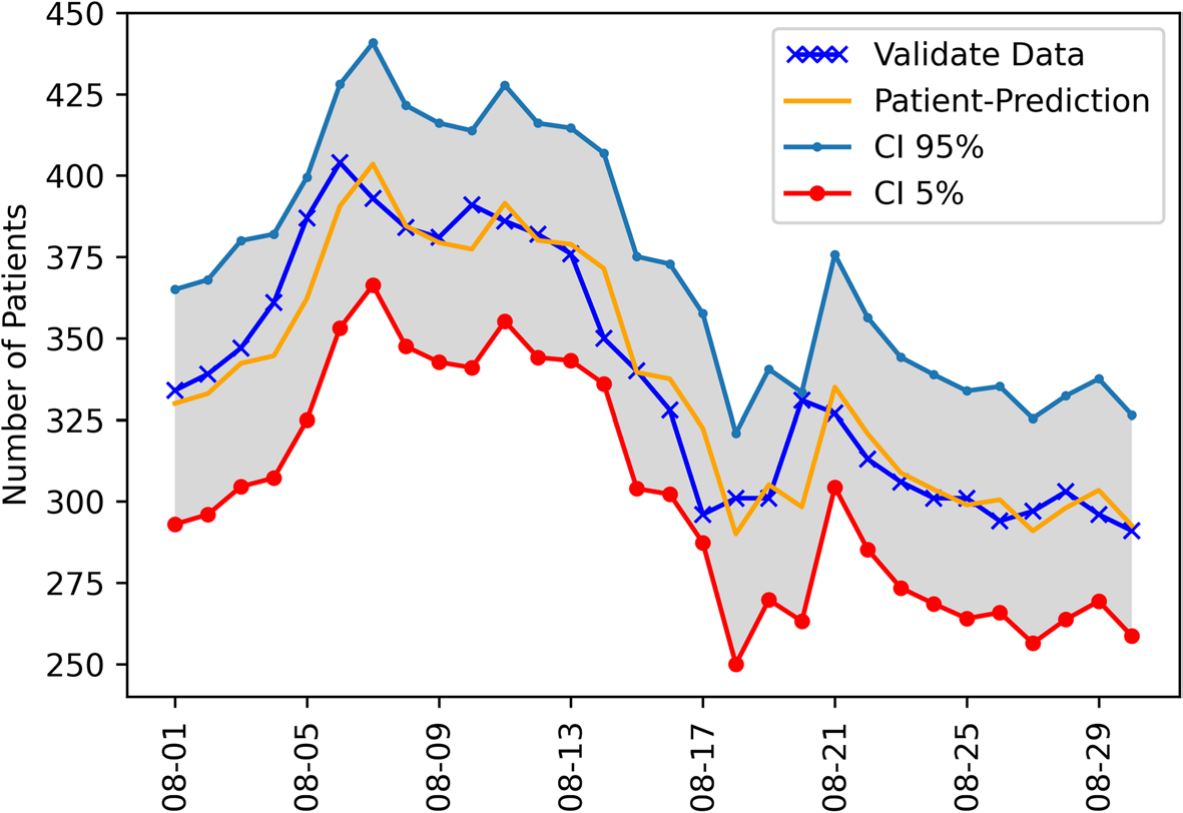
The ARIMAX (1, 1, 1) + X2 forecasting value of the admitted patients with prediction confidential intervals (CI) between 5 and 95%

The forecasting-accuracy metrics of the 51 models summarized on the six datasets and the evaluation of models with the measures of *RMSE* and *MAE* are shown in Table 2. The forecasts for the admitted patients with prediction confidential intervals (CI) between 5 and 95% are presented in Fig. 4 for ARIMA (2,2,2) and Fig. 5 for ARIMAX (1,1,1)+ *x*_2_. Overall, the most accurate estimation was obtained by improving from ARIMA (2, 2, 2) to ARIMAX (1, 1, 1) + *x*_2_ for the training set in Wave 4, covering from 11 April 2021 to 31 May 2021. For the first setting (All-Wave), the best model is ARIMA (1,2,1) with the *RMSE* of 22.8141 and *MAE* of 19.4133, which was closer to the actual data. For Wave-1, ARIMAX (2,2,2) + *x*_2_ performs the best with the *RMSE* of 277.9974 and *MAE* of 273.4644, which was the highest to the actual data of all models. For Wave-2, AR(1) + *X*1 model is the best with the smallest *RMSE* and *MAE*. Based on *RMSE* and *MAE*, the value of ARIMA (1,1,1) + *X*1 was the closest to the actual data in Wave-3. The *RMSE* and *MAE* of ARIMAX (1,1,1)+ *X*2 appeared to be the best predictive models.

**Table 2:** The results of time series analysis model applied to six training sets obtained from statistical tests: Coefficient of determination (R^2^), Root mean square error (RMSE), Mean absolute error (MAE)

No	Model	All Wave			Wave 1			Wave 2			Wave 3			Wave 4			Wave 3–4		
		R2	RMSE	MAE	R2	RMSE	MAE	R2	RMSE	MAE	R2	RMSE	MAE	R2	RMSE	MAE	R2	RMSE	MAE
1	I(1)	0.0899	290.7718	283.8123	0.0331	325.9969	323.9485	0.0904	277.9974	273.4644	0.0328	94.5392	75.6991	0.0145	65.7031	65.7790	0.0522	63.3078	63.8452
2	I(2)	0.2199	188.6716	178.0013	0.2199	325.9572	323.9855	0.2972	285.9000	280.6391	0.0425	77.1222	68.0936	0.0048	83.1201	73.3845	0.0425	80.7247	71.4506
3	AR(1)	0.5835	52.9774	44.8681	0.5172	334.5952	332.3318	0.5186	318.9675	315.8692	0.2275	85.0022	67.6283	0.5543	60.0630	49.5482	0.4259	30.1341	25.6407
4	AR(2)	0.5326	48.9132	45.3217	0.5543	338.2526	336.1396	0.0169	298.2876	295.3547	0.0001	78.0756	66.2715	0.3493	35.2782	26.6954	0.1111	57.0969	46.8206
5	AR(3)	0.5026	71.3773	67.2935	0.5969	337.3307	335.1067	0.1671	292.9912	290.9822	0.0084	82.1250	69.8914	0.1274	53.4205	42.5510	0.1001	61.1195	51.1240
6	MA(1)	0.0004	176.9638	171.7192	0.0004	327.4278	325.3349	0.0004	294.4148	292.0716	0.0004	81.4992	72.4591	0.0004	81.6120	71.7955	0.0004	270.0047	265.8700
7	MA(2)	0.0003	205.0899	199.6313	0.0003	329.3588	327.2778	0.0004	328.9829	326.8977	0.0003	93.4902	85.6784	0.0003	148.8627	141.7412	0.0003	92.1310	83.2050
8	MA(3)	0.0001	307.5673	298.8213	0.0002	328.1374	326.0477	0.0000	333.1808	331.1216	0.0004	149.7222	145.0442	0.0001	125.3216	117.2491	0.0000	79.3442	68.9794
9	ARMA(1,1)	0.5741	40.8981	36.6221	0.5020	334.0945	331.8095	0.4767	315.0413	311.7129	0.1788	85.6014	69.1618	0.5161	42.8637	38.1547	0.3543	33.6863	26.0659
10	ARMA(2,2)	0.5062	67.3449	63.3953	0.6258	339.1666	337.0229	0.0006	299.0722	296.2244	0.5596	203.8326	202.3505	0.1104	56.4891	45.8304	0.0368	72.2012	61.3681
11	ARMA(3,3)	0.5089	68.0282	64.0255	0.6393	334.7573	332.3653	0.0009	306.0231	303.3267	0.1338	147.5160	142.7802	0.1028	56.5914	45.7511	0.3217	62.0183	56.2090
12	ARIMA(1,1,1)	0.4182	121.1567	105.5253	0.0007	327.7731	325.6391	0.3813	275.0749	269.6568	0.2256	61.1524	51.7814	0.5694	43.9619	38.7228	0.7279	35.4740	29.4524
13	ARIMA(2,1,2)	0.4496	188.1384	175.6110	0.1136	331.8481	329.6721	0.0337	274.7054	269.5087	0.5424	241.8948	239.8888	0.0361	60.5829	48.7030	0.8066	37.8168	33.3437
14	ARIMA(3,1,3)	0.5746	100.8429	93.3260	0.0072	327.6217	325.5377	0.1379	291.6125	282.3766	0.0935	145.4436	140.7227	0.0324	60.5233	48.3732	0.2291	99.0573	92.0246
15	ARIMA(1,2,1)	0.6227	22.8141	19.4113	0.0022	327.9731	325.8776	0.5564	307.1235	303.0277	0.1896	85.9017	69.5801	0.5616	43.7555	38.6440	0.3546	33.6537	26.0567
16	ARIMA(2,2,2)	0.5811	108.9374	99.9407	0.0042	330.6365	328.5217	0.0735	280.7723	274.8963	0.5010	221.8999	220.2538	^**a**^**0.5853**	^**a**^**29.7605**	^**a**^**27.6102**	0.0367	78.2477	67.6000
17	ARIMA(3,2,3)	0.5684	105.7827	98.0635	0.0399	328.8342	326.6476	0.0782	269.6121	246.3643	0.7882	147.4570	143.0789	0.1616	85.7303	66.8601	0.3501	81.0317	75.6607
18	I(1) + X1	0.0037	288.6017	283.7774	0.0490	326.2426	324.0655	0.0342	282.0157	279.3011	0.0000	99.6746	75.6989	0.0000	81.8578	72.0013	0.0044	103.9072	83.0929
19	I(2) + X1	0.2592	212.5173	197.9378	0.2592	326.2460	324.1258	0.0005	286.8855	283.5395	0.0019	99.7003	76.0409	0.0048	83.1201	73.3845	0.0068	103.7216	83.5353
20	AR(1) + X1	0.6067	610.2414	519.6976	0.1226	326.8148	324.8728	0.2344	232.7690	219.5232	0.4300	38.4820	32.1837	0.6319	249.6770	198.6695	0.0122	268.9514	263.4326
21	AR(2) + X1	0.5362	67.2016	59.9040	0.1071	327.5698	325.6145	0.0035	263.3728	258.8025	0.6491	36.7028	29.6225	0.3990	88.3833	67.2515	0.8336	100.1792	83.2140
22	AR(3) + X1	0.6796	75.6844	55.5952	0.0890	326.4052	324.4467	0.0017	271.8932	267.0636	0.6816	37.0752	29.5750	0.0373	59.1096	47.5392	0.8428	97.1527	78.2065
23	MA(1) + X1	0.0001	252.3404	248.6892	0.1281	336.3468	334.3429	0.0013	298.9810	296.1156	0.0084	104.0982	76.0860	0.0313	87.0295	76.0244	0.0176	103.1149	81.7565
24	MA(2) + X1	0.0702	221.6587	216.9112	0.2267	335.3732	333.4037	0.1160	336.7430	334.5787	0.0004	75.8666	55.0494	0.0382	134.9549	127.8984	0.0138	88.1465	75.0825
25	MA(3) + X1	0.0005	317.8255	309.4642	0.1913	329.5174	327.5438	0.0001	334.6743	332.6033	0.0060	113.7286	77.5648	0.0139	118.1786	109.4121	0.0008	87.2773	72.8789
26	ARMA(1,1) + X1	0.5916	413.4365	358.2175	0.1774	324.0025	322.1328	0.1541	251.9922	243.5882	0.4516	33.2501	26.5740	0.6405	220.4147	176.9939	0.6106	165.2220	163.3853
27	ARMA(2,2) + X1	0.5949	424.4358	366.5745	0.0723	331.6259	329.4747	0.3369	399.6066	398.4546	0.7118	171.6618	170.5084	0.4607	160.5215	149.9203	0.4951	88.6736	83.4224
28	ARMA(3,3) + X1	0.1833	113.5424	100.8362	0.2160	327.6383	325.7101	0.0107	339.7625	337.6229	0.5940	167.3714	164.8800	0.5863	107.5209	84.9299	0.0044	219.6314	192.1180
29	ARIMAX(1,1,1) + X1	0.4182	183.8188	166.3895	0.1277	322.2422	320.3213	0.2574	267.9366	262.3262	0.5140	45.4581	37.5053	0.5694	43.9619	38.7228	0.7704	83.1827	79.9974
30	ARIMAX(2,1,2) + X1	0.6784	124.4039	99.0967	0.2633	321.7203	319.9461	0.0425	285.4226	281.3559	0.6366	176.3529	174.5772	0.0361	60.5829	48.7030	0.8382	59.1795	47.6580
31	ARIMAX(3,1,3) + X1	0.6510	149.8496	127.6797	0.0302	336.0062	333.9879	0.0007	253.1438	247.7629	0.1191	144.5963	139.6924	0.0324	60.5233	48.3732	0.3812	55.4460	50.4435
32	ARIMAX(1,2,1) + X1	0.2403	143.0063	130.9023	0.1210	322.0322	320.1035	0.2928	278.2316	273.1433	0.4498	49.7481	41.2963	0.5616	43.7555	38.6440	0.7471	79.9917	76.4410
33	ARIMAX(2,2,2) + X1	0.2168	95.7906	88.9073	0.3083	316.7929	315.1248	0.0490	287.2771	283.2256	0.5580	207.7418	206.2698	0.5853	29.7605	27.6102	0.1473	98.7315	80.6377
34	ARIMAX(3,2,3) + X1	0.4787	63.5331	57.3825	0.1452	337.2546	335.2641	0.0016	257.9516	251.6657	0.0026	144.7672	139.9775	0.1616	85.7303	66.8601	0.4676	74.2653	70.1748
35	I(1) + X2	0.0447	289.1389	281.2524	0.0041	326.0347	323.9383	0.0884	278.0144	273.4693	0.0000	99.6750	75.6992	0.0000	81.8175	71.9843	0.0044	103.9073	83.0939
36	I(2) + X2	0.0105	249.4103	231.4904	0.0421	326.0106	323.9672	0.2082	284.7981	279.4427	0.0019	99.6955	76.0373	0.0048	83.2961	73.5082	0.0068	104.7077	83.9761
37	AR(1) + X2	0.6156	255.7645	212.8672	0.5172	334.5952	332.3319	0.1727	302.2914	298.4389	0.0282	69.9752	58.5275	0.6311	249.1627	198.4651	0.5834	41.9315	35.5895
38	AR(2) + X2	0.5858	64.0891	52.3809	0.5543	338.2531	336.1401	0.0007	293.3307	290.4134	0.0131	70.7792	58.5222	0.3373	82.2578	62.6506	0.4830	42.5877	37.2450
39	AR(3) + X2	0.5264	28.4479	22.4192	0.5969	337.3308	335.1069	0.1030	298.6989	296.3389	0.0111	72.7548	60.1599	0.0092	57.7292	46.8582	0.4188	51.2708	45.6610
40	MA(1) + X2	0.0004	191.3896	187.3443	0.0004	327.4278	325.3349	0.0064	303.3572	300.9790	0.0116	81.3063	71.5271	0.0307	98.0537	87.9097	0.1876	275.7095	268.5105
41	MA(2) + X2	0.0003	208.8616	204.3860	0.0003	329.3588	327.2778	0.0071	329.9775	327.9153	0.0164	94.5079	86.0634	0.0638	143.4629	136.7615	0.0215	93.9181	84.8925
42	MA(3) + X2	0.0000	313.7185	303.9636	0.0002	328.3339	326.2458	0.0050	333.2174	331.1693	0.0168	83.1832	74.0123	0.0624	136.5470	125.2288	0.0435	87.0651	77.3405
43	ARMA(1,1) + X2	0.6070	188.6286	158.6611	0.5020	334.0945	331.8095	0.2345	301.7636	297.9054	0.0152	80.3589	66.8892	0.6260	208.8213	169.3105	0.6516	76.7976	69.1291
44	ARMA(2,2) + X2	0.5069	71.3063	67.2141	0.6257	339.1664	337.0227	0.0149	301.8609	298.7972	0.5278	160.9546	157.4370	0.4919	188.1245	174.4431	0.3916	113.9236	106.8338
45	ARMA(3,3) + X2	0.5675	30.4833	28.1090	0.1795	324.3882	322.5190	0.3004	308.7024	303.8821	0.5828	157.7017	154.2446	0.6386	253.0751	196.3018	0.1300	43.9619	38.7226
46	ARIMAX(1,1,1) + X2	0.3531	168.2529	153.0882	0.0929	318.2668	316.3056	0.2369	268.3102	262.7485	0.4671	50.0191	41.8890	^**a**^**0.5695**	^**a**^**27.7508**	^**a**^**23.4642**	0.7704	83.1909	80.0059
47	ARIMAX(2,1,2) + X2	0.6484	174.1887	148.7599	0.2435	324.9496	322.5022	0.0147	269.1468	263.4866	0.6058	198.4915	197.1301	0.0519	59.5415	47.9796	0.8380	60.6156	48.6302
48	ARIMAX(3,1,3) + X2	0.6375	170.1995	146.9692	0.1756	323.9133	322.0434	0.0324	275.3298	266.7312	0.0229	144.4264	139.5740	0.0474	59.5452	47.7400	0.0752	91.5523	75.5956
49	ARIMAX(1,2,1) + X2	0.0823	124.5007	114.6162	0.1758	324.0920	322.2535	0.2380	277.5598	272.5563	0.4500	49.7456	41.2962	0.5460	43.4074	38.4491	0.7471	80.0006	76.4500
50	ARIMAX(2,2,2) + X2	0.6342	40.4552	34.6242	0.0904	277.9974	273.4644	0.0191	270.5990	264.7709	0.5524	217.1144	215.5897	0.0927	85.8890	67.4658	0.8189	72.6105	57.7871
51	ARIMAX(3,2,3) + X2	0.6638	33.3190	25.3416	0.2972	285.9000	280.6391	0.0443	266.4733	258.2639	0.7216	146.7202	142.1839	0.1618	85.7246	66.8524	0.0204	129.1237	118.5442

^a^ the best time series analysis model performance

The comparisons among forecasting models are shown in Tables 3, 4 and 5. The models numbered 12–17 in Table 2 are defined to be the baseline models. The models with *x*_1_ are the models numbered 29–34 while the models with *x*_2_ are the models numbered 46–51. The compared pairs were (baseline vs *x*_1_), (*x*_1_ vs *x*_2_), and (baseline vs *x*_2_). The comparison was done under the same parameter setting. The result of R,^2^ RMSE and MAE (Tables 3, 4 and 5) yielded a good result indicating that time forecasting models could improve correlation of determination when we added exogenous variables.

**Table 3:** The comparison of Coefficient of determination (R^2^)

No	R^2^ Comparison	All Wave		Wave 1		Wave 2		Wave 3		Wave 4		Wave 3–4	
		Win	Loss	Win	Loss	Win	Loss	Win	Loss	Win	Loss	Win	Loss
1	baseline vs *x*_1_	3	3	6	0	1	5	5	1	0	6	6	0
2	*x*_1_ vs *x*_2_	2	4	3	3	2	4	2	4	4	2	3	3
3	baseline vs *x*_2_	4	2	6	0	0	6	4	2	4	2	4	2
	SUM	9	9	15	3	3	15	11	7	8	10	13	5

**Table 4:** The comparison of Root mean square error (RMSE)

No	RMSE Comparison	All Wave		Wave 1		Wave 2		Wave 3		Wave 4		Wave 3–4	
		Win	Loss	Win	Loss	Win	Loss	Win	Loss	Win	Loss	Win	Loss
1	baseline vs *x*_1_	3	3	4	2	4	2	6	0	6	0	2	4
2	*x*_1_ vs *x*_2_	4	2	4	2	3	3	2	4	5	1	1	5
3	baseline vs *x*_2_	3	3	6	0	6	0	6	0	5	1	2	4
	SUM	10	8	14	4	13	5	14	4	16	2	5	13

**Table 5:** The comparison of Mean Absolute error (MAE)

No	MAE Comparison	All Wave		Wave 1		Wave 2		Wave 3		Wave 4		Wave 3–4	
		Win	Loss	Win	Loss	Win	Loss	Win	Loss	Win	Loss	Win	Loss
1	baseline vs *x*_1_	3	3	4	2	3	3	6	0	6	0	2	4
2	*x*_1_ vs *x*_2_	4	2	4	2	3	3	2	4	5	1	1	5
3	baseline vs *x*_2_	3	3	6	0	5	1	6	0	5	1	2	4
	SUM	10	8	14	4	11	7	14	4	16	2	5	13

The predicted values, CI 5% (lower confidence interval) and CI 95% (upper confidence interval), and actual data of the models are shown in Table 6 and Fig. 4. In addition, the improved predictive values of the models by adding exogenous variables are shown in Table 7 and Fig. 5. For example, ARIMA (2, 2, 2) predicted that the number of cumulative confirmed cases for the next 30 days could be 291 to 334 cases. ARIMAX (1, 1, 1) + *x*_2_ predicted that the number of cumulative confirmed cases for the next 30 days could be 293–330 cases.

**Table 6:** The number of patient prediction for time-series model ARIMA (2, 2, 2) + X2 Training from May 1 to July 22, 2021, Prediction from August 1 to August 30, 2021

Date	Actual data	Prediction	Lower CI	Upper CI
August 1, 2021	334	361	327	394
August 2, 2021	339	313	279	347
August 3, 2021	347	326	292	361
August 4, 2021	361	346	311	380
August 5, 2021	387	364	330	398
August 6, 2021	404	395	361	430
August 7, 2021	393	411	377	445
August 8, 2021	384	386	351	420
August 9, 2021	381	371	337	405
August 10, 2021	391	372	338	406
August 11, 2021	386	390	356	424
August 12, 2021	382	381	348	415
August 13, 2021	376	375	342	408
August 14, 2021	350	368	335	401
August 15, 2021	340	335	302	368
August 16, 2021	328	328	295	361
August 17, 2021	296	319	286	352
August 18, 2021	301	280	247	313
August 19, 2021	301	295	262	328
August 20, 2021	331	301	268	334
August 21, 2021	327	342	309	375
August 22, 2021	313	331	297	364
August 23, 2021	306	305	272	338
August 24, 2021	301	299	266	332
August 25, 2021	301	297	264	330
August 26, 2021	294	300	267	332
August 27, 2021	297	291	259	323
August 28, 2021	303	296	264	328
August 29, 2021	296	305	273	337
August 30, 2021	291	294	262	326

*CI* confidence interval

**Table 7:** The number of patient prediction for time-series model ARIMAX (1,1,1) + X2 Training from May 1 to July 22, 2021, Prediction from August 1 to August 30, 2021

Date	Actual data	Prediction	Lower CI	Upper CI
August 1, 2021	334	330	293	365
August 2, 2021	339	333	296	368
August 3, 2021	347	342	305	380
August 4, 2021	361	345	307	382
August 5, 2021	387	362	325	399
August 6, 2021	404	391	353	428
August 7, 2021	393	404	366	441
August 8, 2021	384	385	348	422
August 9, 2021	381	379	343	416
August 10, 2021	391	377	341	414
August 11, 2021	386	392	355	428
August 12, 2021	382	380	344	416
August 13, 2021	376	379	343	415
August 14, 2021	350	371	336	407
August 15, 2021	340	340	304	375
August 16, 2021	328	338	302	373
August 17, 2021	296	322	287	358
August 18, 2021	301	290	250	321
August 19, 2021	301	305	270	341
August 20, 2021	331	298	263	333
August 21, 2021	327	335	304	376
August 22, 2021	313	321	285	356
August 23, 2021	306	309	273	344
August 24, 2021	301	304	269	339
August 25, 2021	301	299	264	334
August 26, 2021	294	301	266	335
August 27, 2021	297	291	256	325
August 28, 2021	303	298	264	332
August 29, 2021	296	303	269	338
August 30, 2021	291	293	259	327

*CI* confidence interval

### Association rule mining

This section explores the association analysis when association rule mining is applied. We present significant rules for the data that included four attributes’ values in the dataset. Table 1 shows preliminary analysis of dataset that was extracted for a total of 3685 patients. The patient data consist of eleven attributes and 35 attribute values. In addition, an attribute code is defined for item set name and frequency of each attribute code. We extract 595 significant rules for the data.

The association rules grouped by four attributes related to managing hospital resources are shown in Table 8. Length of stay more than 14 days is related to healthcare workers and three underlying diseases other, pregnant, and dyslipidemia that have the same value of 1.017. Length of stay less than 14 give the interesting result on symptom mode (Lift of 6.464), three underlying diseases, and age more than 65 years old.

**Table 8:** Top 5 association rules for different combinations of particular consequence, their Support, Average-confidence, Confidence (LHS ➔ RHS), Confidence (RHS ➔ LHS) and Lift measures

No	LHS	RHS	N(A)	N(B)	N(A,B)	Sup_LR_	Conf_A_	Conf_LR_	Conf_RL_	Lift_LR_
Length of Stay less than or equal to 14 days										
1	job_health	los_1_14	93	3625	93	2.524	51.283	100.000	2.566	1.017
2	ud_oth	los_1_14	64	3625	64	1.737	50.883	100.000	1.766	1.017
3	ud_preg	los_1_14	23	3625	23	.624	50.317	100.000	.634	1.017
4	ud_dlp	los_1_14	14	3625	14	.380	50.193	100.000	.386	1.017
5	cxr_pneu	los_1_14	472	3625	470	12.754	56.271	99.576	12.966	1.012
Length of Stay more than or equal to 15 days										
1	sym_mode	los_15	19	60	2	.054	6.930	10.526	3.333	6.465
2	ud_meta	los_15	53	60	3	.081	5.330	5.660	5.000	3.476
3	ud_dm	los_15	18	60	1	.027	3.611	5.556	1.667	3.412
4	ud_ht	los_15	39	60	2	.054	4.231	5.128	3.333	3.150
5	age_65	los_15	74	60	2	.054	3.018	2.703	3.333	1.660
Home Discharge										
1	ud_ht	dc_home	39	3600	39	1.058	50.542	100.000	1.083	1.024
2	ud_dm	dc_home	18	3600	18	.488	50.250	100.000	.500	1.024
3	ud_dlp	dc_home	14	3600	14	.380	50.194	100.000	.389	1.024
4	age_24	dc_home	1148	3600	1131	30.692	64.968	98.519	31.417	1.008
5	cxr_pneu	dc_home	472	3600	465	12.619	55.717	98.517	12.917	1.008
Refer to General hospital										
1	sym_mode	dc_hosp	19	85	4	.109	12.879	21.053	4.706	9.127
2	ud_preg	dc_hosp	23	85	3	.081	8.286	13.043	3.529	5.655
3	ud_meta	dc_hosp	53	85	6	.163	9.190	11.321	7.059	4.908
4	los_15	dc_hosp	60	85	5	.136	7.108	8.333	5.882	3.613
5	age_65	dc_hosp	74	85	6	.163	7.583	8.108	7.059	3.515
Chest X-ray is No lesion										
1	job_health	cxr_no	93	3213	91	2.469	50.341	97.849	2.832	1.122
2	source_hosp	cxr_no	91	3213	88	2.388	49.721	96.703	2.739	1.109
3	age_24	cxr_no	1148	3213	1058	28.711	62.545	92.160	32.929	1.057
4	symp_ast	cxr_no	2295	3213	2112	57.313	78.880	92.026	65.733	1.055
5	ud_repp	cxr_no	82	3213	73	1.981	45.648	89.024	2.272	1.021
Chest X-ray is Pneumonia										
1	sym_mode	cxr_pneu	19	472	8	.217	21.900	42.105	1.695	3.287
2	age_65	cxr_pneu	74	472	31	.841	24.230	41.892	6.568	3.271
3	ud_ht	cxr_pneu	39	472	11	.299	15.268	28.205	2.331	2.202
4	ud_dm	cxr_pneu	18	472	5	.136	14.419	27.778	1.059	2.169
5	ud_meta	cxr_pneu	53	472	14	.380	14.691	26.415	2.966	2.062
Current incidence in Wave 1										
1	los_15	wave_1	60	55	13	.353	22.652	21.667	23.636	14.517
2	source_hosp	wave_1	91	55	16	.434	23.337	17.582	29.091	11.780
3	job_health	wave_1	93	55	15	.407	21.701	16.129	27.273	10.806
4	dc_hosp	wave_1	85	55	6	.163	8.984	7.059	10.909	4.729
5	symp_ast	wave_1	2295	55	54	1.465	50.267	2.353	98.182	1.576
Current incidence in Wave 2										
1	job_health	wave_2	93	311	13	.353	9.079	13.978	4.180	1.656
2	symp_ast	wave_2	2295	311	266	7.218	48.560	11.590	85.531	1.373
3	source_hosp	wave_2	91	311	10	.271	7.102	10.989	3.215	1.302
4	bmi_25_29	wave_2	931	311	96	2.605	20.590	10.311	30.868	1.222
5	bmi_30	wave_2	445	311	42	1.140	11.472	9.438	13.505	1.118
Current incidence in Wave 3										
1	symp_ast	wave_3	2295	1779	1285	34.871	64.111	55.991	72.232	1.160
2	age_25_44	wave_3	1838	1779	1009	27.381	55.807	54.897	56.717	1.137
3	cxr_no	wave_3	3213	1779	1635	44.369	71.396	50.887	91.906	1.054
4	ud_none	wave_3	3392	1779	1700	46.133	72.839	50.118	95.559	1.038
5	bmi_25	wave_3	2309	1779	1136	30.828	56.527	49.199	63.856	1.019
Current incidence in Wave 4										
1	ud_preg	wave_4	23	1540	22	.597	48.540	95.652	1.429	2.289
2	ud_dm	wave_4	18	1540	17	.461	47.774	94.444	1.104	2.260
3	age_65	wave_4	74	1540	64	1.737	45.321	86.486	4.156	2.069
4	ud_meta	wave_4	53	1540	43	1.167	41.962	81.132	2.792	1.941
5	sym_mode	wave_4	19	1540	15	.407	39.961	78.947	.974	1.889

The interesting rule of discharge had two value attributes. The result showed that referral to hospitals was strongly related to symptom of Mode (Lift of 9.127). In addition, four features in this attribute showed high Lift values; underlying diseases (5.655), metabolic syndrome (4.098), length of stay more than 14 days (3.613), and age more than 65 years old (5.515). Chest x-ray with no lesion presented the same level of Lift. However, two features which showed high numbers of patients were age less than 24 years old (1148) and symptom asymptomatic (2295). Moreover, chest x-ray with pneumonia showed all high interesting value Symptom of Mode (3.287), age more than 65 (3.271), underlying diseases diabetes mellitus (2.169), and underlying diseases Metabolic (2.062). In current incident, Wave 1 showed high interest on Length of stay more than 14-days and source of infection from hospital and healthcare worker patients. Wave 2 was also related to healthcare worker, asymptomatic and source of infection from hospital, as was Wave 3. In Wave 4, underlying diseases, age more than 65 and symptom mode showed strong relationships. Association rules selected key attributes of the data set to be exogenous variables of a time series analysis.

## Discussion

The first wave of SARS-CoV-2 occurred in early 2020, and the second, third and fourth waves rapidly spread from early to mid-2021, representing an unprecedented phenomenon in medical services, society and the economy of Thailand. The number of COVID-19 patients shown in this study increased from the first wave of just 55 patients to 311, 1779 and 1540 in the second, third and fourth waves, respectively, which evolved more than 30 times of the total number of patients admitted at the field hospital. Most of patients were at least 44 years old and were predominantly female. Patients included in this study were mostly asymptomatic and had no sign of pneumonia in the chest x-ray due to the field hospital system’s focus on patients who did not require advanced treatment. But during the third and fourth waves, the number of mild to moderate symptoms with pneumonia of COVID-19 patients significantly increased because of the greater severity of the delta variant of SARS-COV-2. The huge number of patients was a burden on the limited resources of Thailand’s healthcare system. Therefore, this study presented the use of time series modeling and association rule mining to forecast the COVID-19 pandemic outbreak as well as to analyze its associated prognostic factors. The method presented a data-oriented approach that applies time-series analysis and association analysis to reveal meaningful hidden patterns for efficient handling of another pandemic crisis.

ARIMA models have been successfully applied for predicting the disease outbreak. Several studies have utilized the ARIMA model to forecast the spread of COVID-19 in many countries including the US, Brazil, India, Russia and Spain [28, 29]. The studies using ARIMA models to predict COVID-19 cases relative to total confirmed cases presented an average RMSE of 144.81 across 6 geographic regions [28], MAE of 787 to 1506 in USA and 82 to 570 in Italy [18], and MAE of 2967 in Indonesia [20]. In this work, ARIMA (2, 2, 2) was selected as the most accurate ARIMA model for predicting the number of admitted COVID-19 cases in the field hospital, which achieved a R^2^ = 0.5695, RMSE = 29.7605, MAE = 27.5102 (Fig. 4). The forecast results of admitted cases on August 15 and August 30, 2021 were 335 and 294, respectively. In comparison with the actual values reported on the same dates, the forecasted values of our selected ARIMA model were within the upper and lower bounds at 95% confidence intervals. This signified an acceptable accuracy of this model for estimating admitted cases in the field hospital.

ARM is a structured method of discovering frequent patterns in a data set and forming noticeable rules among regular patterns. In the COVID-19 crisis, many nations, including Thailand, have a highest priority to save lives and protect their economies. A previous study using ARM for mining COVID-19 data to analyze factors related to COVID-19 situation management showed that face mask mandates combined with mobility reduction through moderate stay-at-home orders were most effective in reducing the number of COVID-19 cases in United State [24]. In this study, the ARM technique was used to analyze and identify factors related to the length of stay and prognosis of COVID-19 patients and found that the top five factors related to hospital stays longer than 14 days consisted of healthcare workers uncommon underlying diseases such as thalassemia, thyroid diseases, gout and G6PD deficiency, pregnant patients, dyslipidemia and signs of pneumonia in chest x-rays. This study also identified a clinical factor rule related to the worsening condition of the inpatient. Among those who needed more advanced medical treatment, the rules included mild to moderate COVID-19 symptoms, pregnant patients, metabolic syndrome, length of hospital stay more than 14 days, and patients older than 65 years old. These factors are consistent with those in a previous study, which reported similar conditions among patients who had a poor prognosis in COVID-19 infections [1, 30].

In any prediction tasks, more data is needed to achieve better performance from the models. This study developed the combination of the ARM technique and the ARIMA model, as the ARIMAX model. This model worked by selecting the rules related to COVID-19 prognosis from the ARM technique, including mild to moderate COVID-19 symptoms, patients with metabolic syndrome and patients older than 65 years old, and integrating them to the ARIMA model. Experimental results showed that the ARIMAX model (1, 1, 1) improved the accuracy of forecasting the number of admitted COVID-19 cases, which achieved a *R*^2^ = 0.5695, RMSE = 27.7508, MAE = 23.4642 (Fig. 5). The forecast value of this model for August 30, 2021 was estimated to be 259 to 327 cases. The actual number of cases on the same date was 291 cases. The actual value also was within the lower and upper prediction bounds for both 95% confidence intervals. To the best of our knowledge, this is the first study to combine the ARM technique with the ARIMA model for forecasting the COVID-19 cases by integrating the optimal exogenous variables from the ARM rules to form a predictive model. This ARIMAX model had the potential to predict the number of COVID-19 patients, which could be one of the reliable forecasting-based models for the future outbreak. These predictive models are intended to help better decision-making to plan an effective management system if the virus outbreak has not subsided.

### Limitations

The limitation of this study is that the dataset was based on retrospective data from a single COVID-19 field hospital in Thailand with a limited number of cases and clinical variables of COVID-19 patients.

### Future directions

In future work, the collaboration between multi-medical centers for a larger number and different variables of COVID-19 cases, including the medical records of clinical, laboratory and treatment data from various COVID-19 centers, would upgrade the forecasting performance of this AI model to predict the COVID-19 event more accurately. Additionally, geographic data related to the pandemic area could be used as a variable for alternative time series models such as space-time ARIMA models [31], which could be more reliable in predicting future COVID-19 outbreaks.

## Conclusion

This study demonstrated that the ARIMAX model has the potential to increase the accuracy for predicting the number of COVID-19 cases by incorporating the most associated prognostic factors identified by ARM technique to the ARIMA model. The result of this study proved to be an effective AI model to predict the number of and to identify prognostic factors of admitted COVID-19 patients. This work is expected to be a novel AI-based decision-making model for preparation, organizing hospital resources and more optimal use of medical personnel and equipment to enhance healthcare decision-making, and to manage the COVID-19 pandemic but as well as other epidemic crises.

## Data Availability

The datasets used and/or analyzed during the current study are available from the corresponding author on reasonable requests.

## Abbreviations

COVID-19: Coronavirus disease 2019
SARS-CoV-2: Severe Acute Respiratory Syndrome-*Coronavirus-2*
MERS-CoV: Middle East Respiratory Syndrome Coronavirus
CBC: Complete blood count
LFTs: Liver function tests
BUN: Balance urine nitrogen
Cr: Creatinine
SpO_2_: Pulse oxygen saturation
BT: Body temperature
BMI: Body mass index
G6PD: Glucose-6-Phosphate Dehydrogenase
ANN: Artificial Neural Network
SVM: Support Vector Machine
ARM: Association Rule Mining
ARIMA: Auto Regressive Integrated Moving Average
ARIMAX: Autoregressive Integrated Moving Average with Exogenous Covariates
R^2^: Coefficient of determination
RMSE: Root mean square error
MAE: Mean absolute error
CI: Confidence intervals
